# Electrochemical deposition of amorphous cobalt oxides for oxygen evolution catalysis†

**DOI:** 10.1039/d2ra00492e

**Published:** 2022-03-21

**Authors:** Wei Liu, Masao Kamiko, Ikuya Yamada, Shunsuke Yagi

**Affiliations:** Institute of Industrial Science, The University of Tokyo 4–6–1 Komaba, Meguro-ku Tokyo 153–8505 Japan syagi@iis.u-tokyo.ac.jp; Department of Materials Science, Graduate School of Engineering, Osaka Prefecture University 1–1 Gakuen-cho, Naka-ku, Sakai Osaka 599–8531 Japan

## Abstract

The oxygen evolution reaction (OER) is crucial in water splitting for hydrogen production. However, its high over-potential and sluggish kinetics cause an additional energy loss and hinder its practical application. The cobalt spinel oxide Co_3_O_4_ exhibits a high catalytic activity for the OER in alkaline solutions. However, the activity requires further enhancement to meet the industrial demand for hydrogen production. This paper presents an electrochemical deposition method to obtain cobalt oxides with a controllable crystallinity on carbon paper (CP). Usually, cobalt oxides grown on CP have a Co_3_O_4_ spinel oxide structure. The self-supported Co_3_O_4_/CP exhibited a considerable catalytic activity for the OER. When a VS_2_ layer grown on the CP beforehand by a hydrothermal method was used as substrate, the deposited cobalt oxides were in an amorphous state, denoted as CoO_*x*_/VS_2_/CP, which exhibited a higher OER activity and better stability than those of Co_3_O_4_/CP. The enhancement in the catalytic activity was attributed to the mixture formation of different types of cobalt species, including Co_3_O_4_, CoO, Co(OH)_2_, and metallic Co, because of the reduction by VS_2_. We also clarify the significance of the crystallinity of cobalt oxides in the improvement in the OER activity. This process can also be applied to the direct formation of other types of self-supported oxide electrodes for OER catalysis.

## Abstract 2

Amorphous cobalt oxides electrodeposited on VS_2_ grown on carbon paper show better catalytic activity for the oxygen evolution reaction than crystalline Co_3_O_4_ on carbon paper.

## Graphical abstract

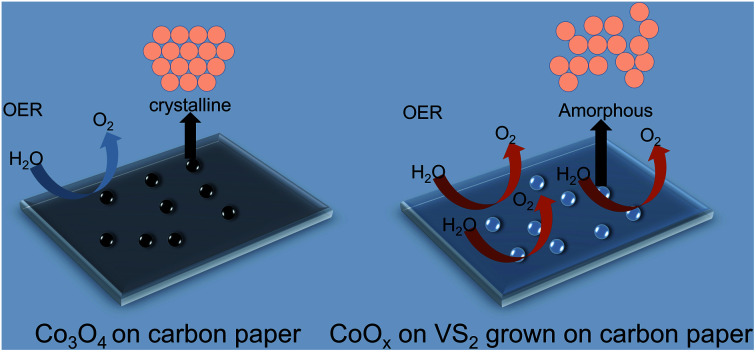

## Introduction

Water electrolysis is a crucial approach for the production of a very large amount of hydrogen, which is a promising alternative energy source to fossil fuels.^1^ Hydrogen evolution reaction proceeds on the cathode during water electrolysis, while oxygen evolution reaction (OER) proceeds on the anode. Owing to multi-step reactions, the OER suffers from a large over-potential and slow kinetics, which lead to a very large energy loss.^2^ Therefore, noble metals and their oxides are commonly used as commercial catalysts to promote OER. However, their high cost hinders the reduction in the cost of hydrogen mass production.^3,4^ Therefore, affordable catalysts with high OER activities are required to reduce the cost of hydrogen production.^5–8^

3d transition-metal oxides, as promising candidates, exhibit high OER activities. However, their activity still cannot meet the demand for industrial production of hydrogen. Extensive studies have been carried out to enhance and analyze the catalytic activities of 3d transition-metal oxides for the OER.^9–12^ For example, Mefford *et al.* observed the surface of Co(OH)_2_ under OER conditions using operand microscopy and reported that the OER proceeds mainly at the edge facets of Co(OH)_2_.^13^ The edges exhibited a higher OER activity than that at the grain interior of Co(OH)_2_. Koza *et al.* synthesized both crystalline and amorphous cobalt oxides by an electrodeposition method and demonstrated that the amorphous cobalt oxide exhibited a higher catalytic activity than that of the crystalline Co_3_O_4_.^14^ Therefore, the amorphization of cobalt oxides is expected to yield a higher activity, but the mechanism is still unclear.

On the other hand, modification with sulfides reduces the resistance in OER catalysis of transition-metal oxides. For example, Kim *et al.* reported that a Co_3_O_4_/MoS_2_ heterostructure exhibited a considerably higher OER catalytic activity than that of the simple Co_3_O_4_.^15^ They reported that Co_3_O_4_ and MoS_2_ underwent electron transfer at the heterogeneous interface, modulating the adsorption energy of oxygen-containing species on the oxide surface, which led to an OER enhancement. Jiang *et al.* reported that a double exchange interaction in the spinel oxide structure was ignited by constructing a heterostructure of MoS_2_ and Fe-doped NiCo_2_O_4_.^16^ Both oxygen vacancies and electronic state transitions activated this effect through the heterostructure between the oxide and sulfide. These heterostructure materials can be synthesized by various methods, such as sol–gel synthesis, solid-state synthesis, electrodeposition, and coprecipitation synthesis.^17–19^ Among them, electrodeposition can be used to precisely adjust the catalyst loading mass on the substrate surface by controlling the electric quantity. It is also ideal for construction of heterostructures between oxides and sulfides because the electrodeposition can achieve a strong adsorption between the two phases by electrostatic adsorption as well as electrochemical reactions.

Therefore, in this study, to combine the above two positive effects, *i.e.*, amorphization and combination with sulfides, we demonstrate the synthesis of amorphous cobalt oxide layers by forming a VS_2_ layer on a carbon paper (CP) beforehand. Unlike the flat surface of the CP, the layered structure of VS_2_ hindered the crystallization of the Co oxides. As a result, amorphous cobalt oxides were deposited on the CP with VS_2_, denoted as CoO_*x*_/VS_2_/CP. CoO_*x*_/VS_2_/CP exhibited an enhanced catalytic activity and better stability for OER than that of Co_3_O_4_/CP. The mechanism of the enhancement is discussed in detail.

## Results and discussion

**Fig. 1:**
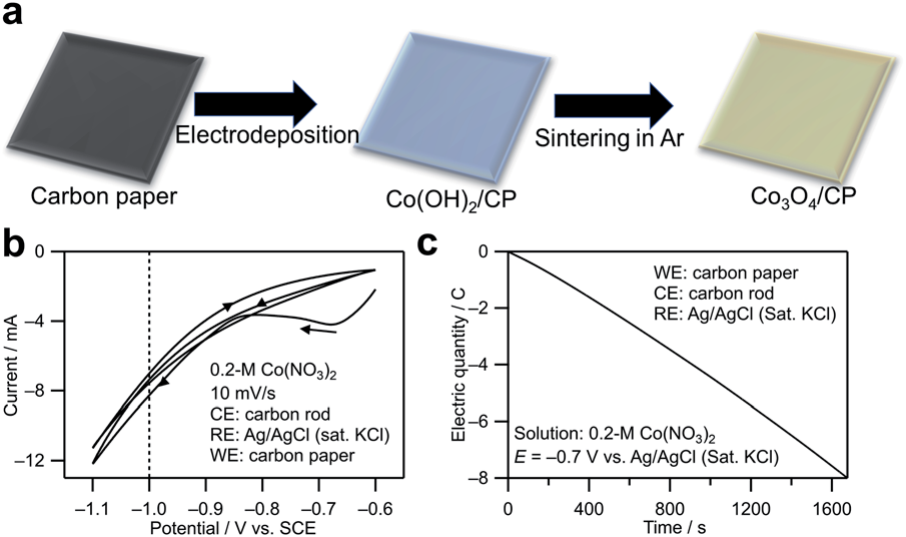
(a) Illustration of the formation of cobalt oxide on the CP. Co(OH)_2_ on carbon paper was obtained initially by electrodeposition in a Co(NO_3_)_2_ aqueous solution, and Co_3_O_4_ on carbon paper was obtained by sintering the deposited Co(OH)_2_ on carbon paper. (b) Cyclic voltammogram and (c) chronocoulogram measured using the CP as the working electrode in the 0.2 M Co(NO_3_)_2_ aqueous solution (pH ∼6).

The synthesis method for cobalt oxide on the CP is shown in Fig. 1a. In the electrodeposition step, the following reactions occur on the cathode (CP):^20^

1 NO^−^_3_ + H_2_O + 2e^−^ → NO^−^_2_ + 2OH^−^,

2 Co^2+^ + 2OH^−^ → Co(OH)_2_

The redox potential of reaction 1 is calculated to be 0.82 − 0.05916 pH (0.47 V *vs.* standard hydrogen electrode (SHE) at pH = 6).^21^Fig. 1b shows the cyclic voltammogram measured using the CP as the working electrode in a 0.2 M Co(NO_3_)_2_ aqueous solution (pH ∼6). The NO_3_^−^ reduction reaction contributed to the cathodic current. Based on the cyclic voltammogram, −0.7 V *vs.* Ag/AgCl (saturated KCl) with a relatively small current was chosen to achieve a steady deposition rate. The reason is that a smaller current is related to a slower deposition rate in the electrodeposition, which is beneficial to the growth of crystal nuclei instead of the formation of nucleation process and is also preferable in chrono coulometry because it is easier to control the electronic quantity precisely by a manual method. Fig. 1c shows a chronocoulogram obtained during electrodeposition (see the related chronoamperogram in Fig. S1†). The deposition amount can be controlled by controlling the transferred electric quantity. In this study, the Co deposition amount was distinguished from the electric quantity transferred during electrodeposition. For example, Co_3_O_4_/CP (0.1C) indicates that electrons with a charge of 0.1C were transferred during the electrodeposition.

**Fig. 2:**
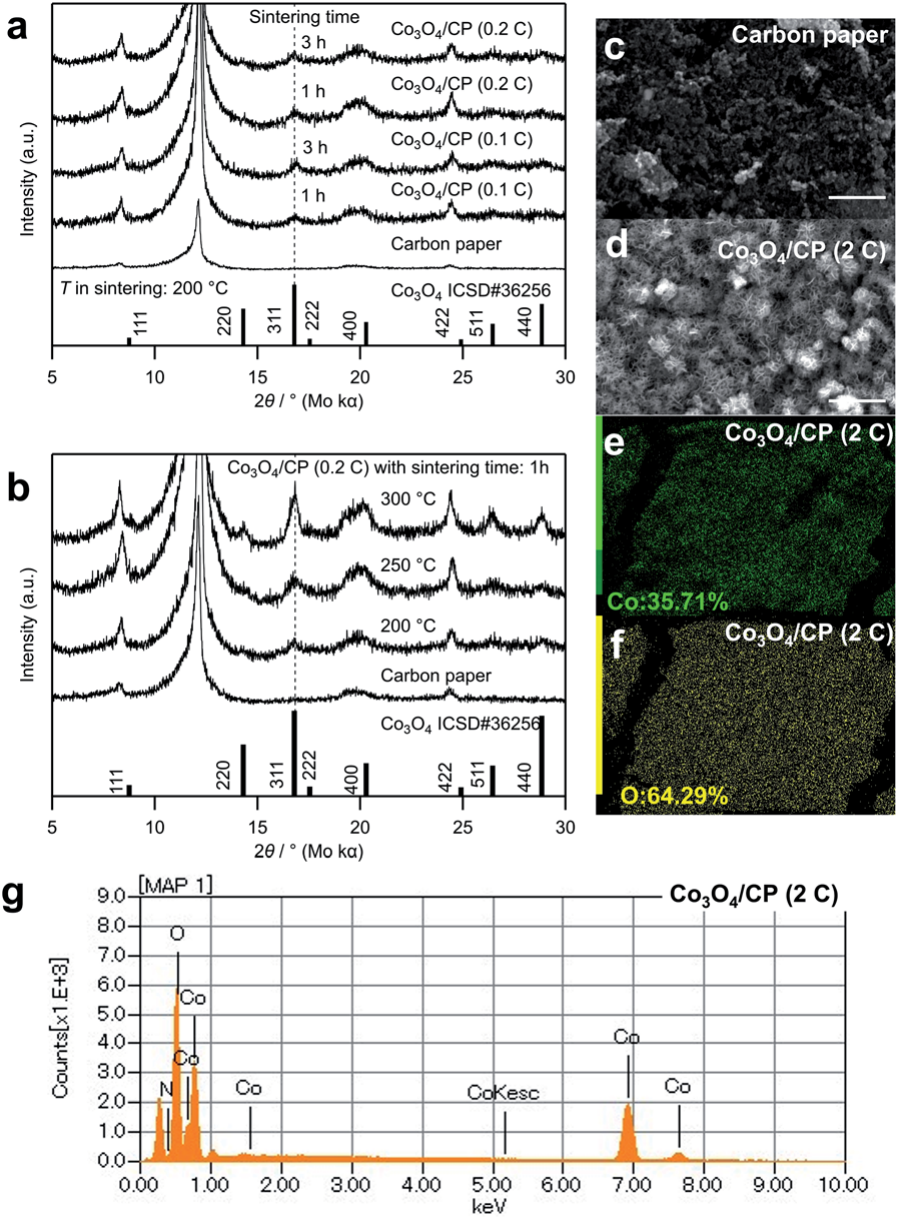
(a) XRD patterns of Co_3_O_4_/CP samples with different deposition amounts and sintering times. The wavelength of Mo Kα is 0.7107 Å. (b) XRD patterns of Co_3_O_4_/CP samples obtained at different sintering temperatures. SEM images of (c) CP and (d) Co_3_O_4_/CP (2C). The scale bar corresponds to 5 μm. (e) Co and (f) O ratios obtained by mapping and (g) spectrum of Co_3_O_4_/CP (2C) determined by EDS.

The crystal structure of the cobalt oxides deposited on the CP was characterized by XRD, as shown in Fig. 2a and b. The peaks of the deposited cobalt oxides correspond to the peaks of the reported crystalline Co_3_O_4_ (ISCD#36256, see 311 at 16.8°).^22^ Notably, there are several peaks attributed to the CP. According to Table S1,† the full width at half maximum (FWHM) of the peak at 16.8° decreased (*i.e.*, the crystallinity of the deposited Co_3_O_4_ increased) with the increase in the electrodeposition amount, sintering temperature, and time. Fig. 2c and d show SEM images of the CP before and after the electrodeposition of cobalt oxides. In Fig. 2d, mesh structure was formed on the surface of the carbon paper. Compared with the structure of the carbon paper, it can be confirmed that these structures are deposited cobalt oxides with the particle size at about 2 μm. The EDS elemental mapping of cobalt and oxygen in Fig. 2e and f and EDS spectrum in Fig. 2g also suggest the existence of cobalt oxides, which indicates a homogeneous deposition.

**Fig. 3:**
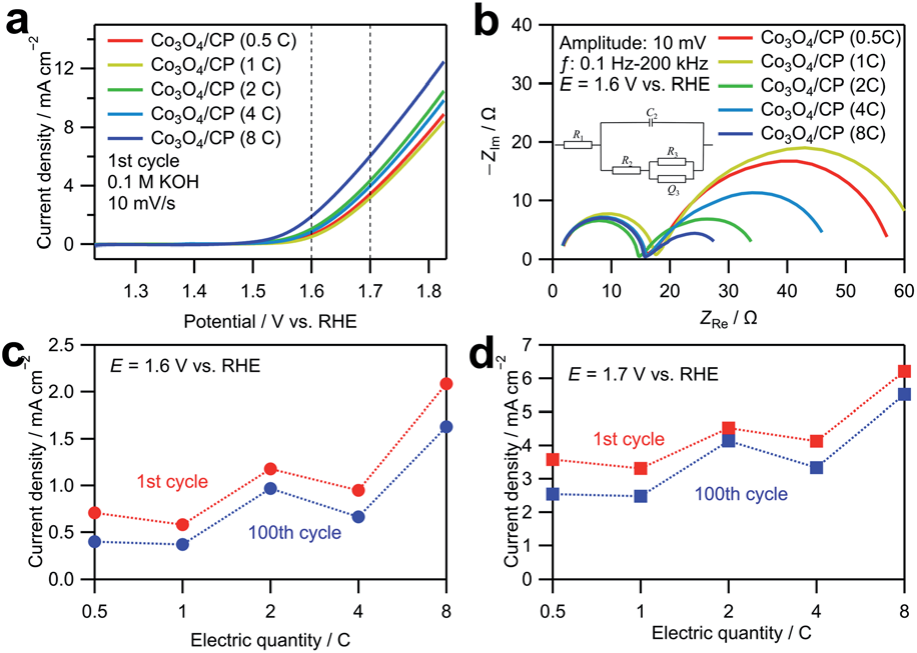
(a) Linear sweep voltammograms obtained by averaging currents during anodic and cathodic sweeps in cyclic voltammetry in the range of 1.23 to 1.83 V *vs.* RHE and (b) Nyquist plots of EIS spectra measured for the Co_3_O_4_/CP samples with different Co deposition amounts at the voltage amplitude of 10 mV in the range of 0.1 Hz to 100 kHz. The cyclic voltammetry was carried out in an O_2_-saturated 0.1 M KOH aqueous solution at a scan rate of 10 mV s^−1^. Current densities of the Co_3_O_4_/CP samples with different Co deposition amounts at (c) 1.6 V and (d) 1.7 V in the first cycle and 100th cycle acquired by CV-averaged linear sweep voltammetry for 100 cycles in the range of 1.23 to 1.83 V *vs.* RHE.

The as-deposited Co_3_O_4_/CP was directly used as an anode in a water electrolysis system without additional modifications. Fig. 3a shows linear sweep voltammograms obtained by averaging the cyclic voltammograms measured for the Co_3_O_4_/CP samples with different Co deposition amounts in an O_2_-saturated 0.1 M KOH aqueous solution to cancel out the capacitive current. Co_3_O_4_/CP used for the OER evaluation was synthesized by annealing at 300 °C for 3 h; only the amount of deposited Co was different. The current densities at 1.6 and 1.7 V *vs.* RHE (dashed lines in Fig. 3a) increase with the increase in the electrodeposition amount of Co_3_O_4_. To investigate this effect, EIS was carried out for all samples at 1.6 V *vs.* RHE. Nyquist plots of the obtained EIS spectra are presented in Fig. 3b. For the semicircle in high frequency range, charge transferring is the main factor that affects the impedance. After the modification of the carbon paper for all the samples, the size of this semicircle did not change so much. Therefore, the semicircles in the high-frequency range resulted from the charge transfer process between the carbon paper and the electrolyte. The other semicircles in the low-frequency range came from the adsorption/desorption process of OER intermediates.^23^ Curve fitting was carried out with the equivalent circuit shown in Fig. S2; † the fitted parameters are listed in Table S2.† The system resistance *R*_1_ did not change significantly among the samples. The charge transfer resistance *R*_2_ and double-layer capacitance *C*_2_ did not significantly change among the samples. The close values of double layer capacitance indicate that all the samples have similar electrochemical surface area, which excludes the influence of surface area difference on OER activity. *R*_3_ and *Q*_3_ are related to the impedance in the adsorption/desorption process of OER intermediates on the catalyst surface. The value change is correlated with the increase in the electrodeposition amount of Co_3_O_4_. Therefore, the enhancement in OER activity is mainly associated with the increase in the diffusion rate of the reactants, which is likely caused by the increased number of active sites with a larger deposition amount. Fig. 3c and d show the current densities of the Co_3_O_4_/CP samples with different Co deposition amounts at 1.6 and 1.7 V *vs.* RHE, respectively. The current densities of all samples similarly decreased after 100 cycles, which indicate similar stabilities.

**Fig. 4:**
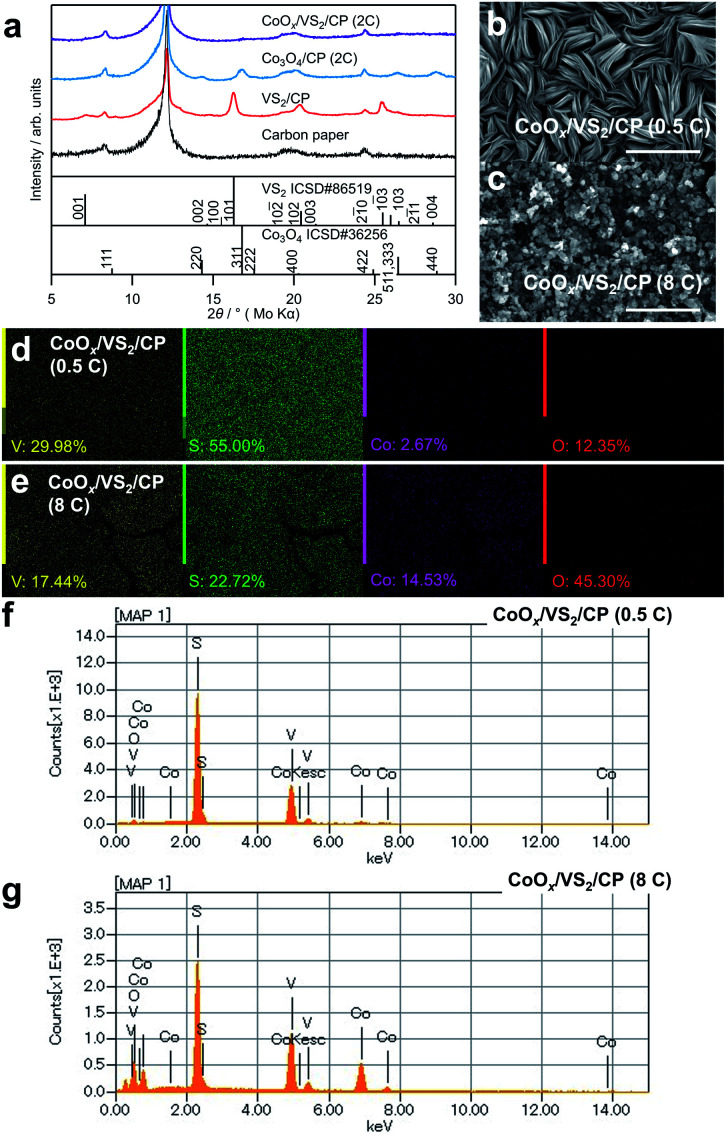
(a) XRD patterns of CoO_*x*_/VS_2_/CP (2C), Co_3_O_4_/CP (2C), VS_2_/CP, and CP (b and c) SEM images of CoO_*x*_/VS_2_/CP (0.5C) and CoO_*x*_/VS_2_/CP (8C) (d and e) EDS elemental ratios and (f and g) spectra of CoO_*x*_/VS_2_/CP (0.5C) and CoO_*x*_/VS_2_/CP (8C).

**Fig. 5:**
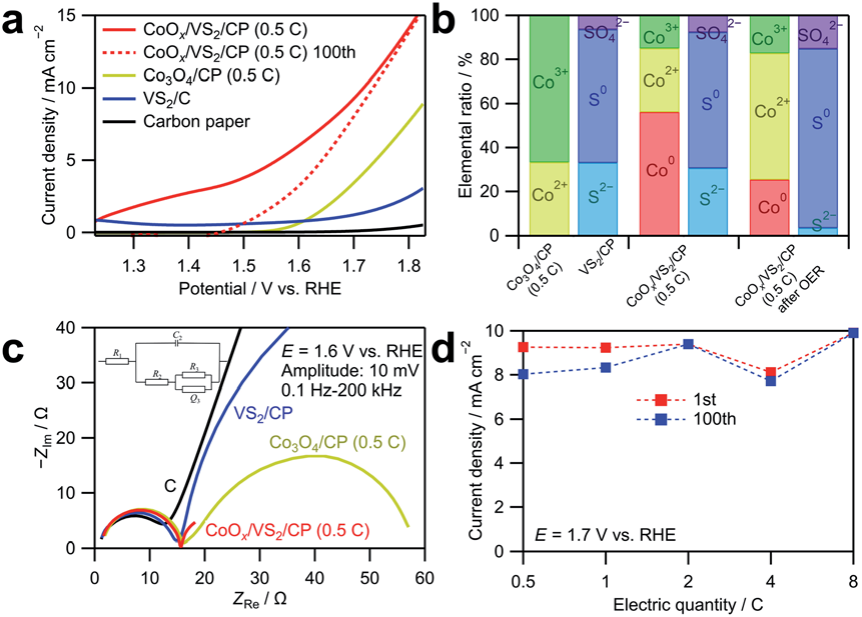
(a) Linear sweep voltammograms obtained by averaging currents during anodic and cathodic sweeps in cyclic voltammetry in the range of 1.23 to 1.83 V *vs.* RHE for CoO_*x*_/VS_2_/CP (0.5C), Co_3_O_4_/CP (0.5C), VS_2_/CP, and CP. The cyclic voltammetry was carried out in an O_2_-saturated 0.1 M KOH aqueous solution at a scan rate of 10 mV s^−1^. (b) Ratios of Co and S with different valence states in Co_3_O_4_/CP (0.5C), VS_2_/CP, and CoO_*x*_/VS_2_/CP (0.5C) before and after the cyclic voltammetry for 100 cycles in the range of 1.23 to 1.83 V *vs.* RHE at a scan rate of 10 mV s^−1^, determined by XPS. (c) Nyquist plots of EIS spectra measured for CoO_*x*_/VS_2_/CP (0.5C), Co_3_O_4_/CP (0.5C), VS_2_/CP, and CP under 1.6 V *vs.* RHE at the voltage amplitude of 10 mV in the range of 0.1 Hz to 100 kHz. (d) Current densities of the CoO_*x*_/VS_2_/CP samples with different Co deposition amounts at 1.6 V in the first cycle and 100^th^ cycle acquired by CV-averaged linear sweep voltammetry for 100 cycles in the range of 1.23 to 1.83 V *vs.* RHE.

To further increase the catalytic activity and stability of cobalt oxides, VS_2_ was grown beforehand on the CP to change the morphology and crystallinity of the cobalt oxides. Fig. 4a shows XRD patterns of the synthesized samples, denoted as CoO_*x*_/VS_2_/CP. Notably, no considerable peaks corresponding to VS_2_ and Co_3_O_4_ are observed in the XRD patterns of CoO_*x*_/VS_2_/CP (2C) in Fig. 4a and CoO_*x*_/VS_2_/CP (8C) in Fig. S3,† although the EDS elemental mapping indicates the existence of V, S, and Co. This is contrary to the pattern of Co_3_O_4_/CP (2C), where the peaks for Co_3_O_4_ were observed. Therefore, the absence of cobalt oxide peaks was not attributed to the deposition of cobalt oxides, but to the existence of VS_2_. As shown in Fig. 4b, CoO_*x*_/VS_2_/CP (0.5C) exhibits a layer morphology of VS_2_ even though Co is evenly distributed on the surface, as shown in Fig. 4d (see gradual morphology in CoO_*x*_/VS_2_/CP with Co deposition amount increase in Fig. S4†). The SEM image of VS_2_ on carbon paper is also displayed in Fig. S4.† The bulks of VS_2_ with a diameter of about 5 μm can be observed. In each bulk, there exists the characteristic layered structure of VS_2_. Thus, it is possible that the cobalt hydroxide precursor was deposited by electrodeposition between the layers of VS_2_. In other words, the VS_2_ layers hinder the generation of large bulk cobalt oxides, resulting in amorphous cobalt oxides after sintering. In addition, the peaks of VS_2_ disappeared, although V and S were detected by EDS mapping (Fig. 4d and e) and EDS spectra in Fig. 4f and g. The slight increase in the background of the XRD pattern for CoO_*x*_/VS_2_/CP (2C) indicates the formation of amorphous phases, as shown in Fig. S5.† The XPS spectra in Fig. S6† also suggest that VS_2_ and cobalt oxides reacted (further evidence is shown in Fig. 5b).

The OER catalytic activity of CoO_*x*_/VS_2_/CP was evaluated by cyclic voltammetry and EIS, as shown in Fig. 5. Before OER measurements, pre-scanning at 1.23–1.83 V *vs.* RHE at 10 mV s^−1^ for 20 cycles was carried out to reach a steady state. The anodic and cathodic sweeps of the cyclic voltammograms were averaged to eliminate the influence of the double-layer capacitance. As shown in Fig. 5a, the current density of CoO_*x*_/VS_2_/CP (0.5C) is considerably higher than those of the other samples. Notably, at the potential range of 1.2 to 1.4 V *vs.* RHE, the current density of CoO_*x*_/VS_2_/CP (0.5C) is too high to be neglected, which should not be attributed to the currents of the OER because the OER does not occur below 1.23 V *vs.* RHE. To investigate this phenomenon, the linear sweep voltammogram of CoO_*x*_/VS_2_/CP (0.5C) at the 100^th^ cycle is also presented in Fig. 5a (red dashed line). The high current density in the low potential range of 1.2 to 1.43 V *vs.* RHE decreased to almost 0 after 100 cycles, which indicates that the oxidation reaction is irreversible. The cyclic voltammograms and Nyquist plot of EIS spectra at 100th cycle of VS_2_ are displayed in Fig. S8.† Compared to the considerable current density in 1st cycle, the current density in 100th cycle in the potential range of 1.2 to 1.5 V shows an obvious decrease, which is due to the irreversible oxidation of S^2−^ ions. However, the oxidation current density of VS_2_/C is small and cannot reach the high current density of CoO_*x*_/VS_2_/CP (0.5C) from 1.2 to 1.5 V in Fig. 5a. Therefore, the high current density of CoO_*x*_/VS_2_/CP (0.5C) from 1.2 to 1.5 V was attributed mainly to the Co oxidation in CoO_*x*_, but not to S^2−^ oxidation in VS_2_. This was also confirmed by XPS, as shown in Fig. 5b (see the deconvoluted XPS spectra in the Co 2p_3/2_ region in Fig. S7†).^24,25^ Before the cyclic voltammetry, Co^0^, Co^2+^, and Co^3+^ existed in CoO_*x*_/VS_2_/CP (0.5C). It is possible that Co^0^ was generated by the reduction of cobalt oxides or hydroxides with S^2−^ ions during the sintering. After the 100 voltammetry cycles, the ratio of Co^0^ decreased, while that of Co^3+^ ions increased, which indicates that the Co oxidation contributes largely to the current at a low OER potential. The comparison of the voltammograms of CoO_*x*_/VS_2_/CP (0.5C) after 100 cycles and Co_3_O_4_/CP (0.5C) in the first cycle shows that CoO_*x*_/VS_2_/CP (0.5C) containing the amorphous CoO_*x*_ exhibited a higher catalytic activity than that of Co_3_O_4_/CP (0.5C) containing the crystalized Co_3_O_4_.

The Nyquist plots of the EIS spectra at 1.6 V *vs.* RHE in Fig. 5c show that all samples exhibit similar behaviors in the higher frequency range. However, the behavior in the lower frequency range is quite different. CoO_*x*_/VS_2_/CP (0.5C) exhibits the smallest semicircle among all samples, which indicates fastest diffusion. To make a comparison, the Nyquist plot of CoO_*x*_/VS_2_/CP (0.5C) at the 100th cycle are also displayed in Fig. S9.† Compared to the 1st cycle, the semicircle in the low frequency is smaller, indicating a faster adsorption/desorption process. Furthermore, curve fitting was carried out for Nyquist plots in Fig. 5c using the equivalent circuit in Fig. S2; † the results are listed in Table S3.†*R*_1_, *R*_2_, and *C*_2_ did not change significantly for all samples in Fig. 5c, but the adsorption resistance *R*_3_ decreased to a large extent for CoO_*x*_/VS_2_/CP (0.5C) compared to those of the other samples. The smallest *R*_3_ and largest *Q*_3_ of CoO_*x*_/VS_2_/CP (0.5C) indicate the fastest adsorption/desorption process and the most reaction sites on the catalyst surface, respectively.

Fig. 5d shows the current densities of the CoO_*x*_/VS_2_/CP samples with different Co amounts at 1.7 V in the first cycle and 100^th^ cycle. The currents are well retained after 100 cycles, compared to the currents of Co_3_O_4_/CP shown in Fig. 3d. This indicates that the amorphous cobalt oxides deposited on VS_2_/CP exhibit a better stability in the OER catalysis, likely because the amorphous material does not have grain boundaries that can react or easily dissolve.

By comparing the data in Fig. 3d and 5d, we can see that the electrodeposition time of cobalt has a positive-related effect on the OER activity of Co_3_O_4_/CP, but barely affects the OER activity of CoO_*x*_/VS_2_/CP. For Co_3_O_4_/CP, the deposited cobalt oxides are crystalized Co_3_O_4_ structure, which was confirmed by XRD patterns. With the electron quantity in the electrochemical deposition increase, the amount of Co_3_O_4_ increases. Co_3_O_4_ plays as the active species in the OER catalysis. Therefore, the amount of Co_3_O_4_ increase indicates an increase of the active species, which will result in an obvious increase of current density for OER. For CoO_*x*_/VS_2_/CP, the deposited cobalt oxides are amorphous, where Co exists as Co^0^, Co^2+,^ and Co^3+^ at the same time from XPS analysis. The increase in deposition time is not directly related to the active species in OER. Increasing deposition time can also increase the number of cobalt oxides, but the deposition time and active species are not directly related. Thus, an increase in current density can be observed by increasing the deposition time for CoO_*x*_/VS_2_/CP, but it is not as obvious as that for Co_3_O_4_/CP.

## Experimental

### Synthesis of cobalt oxides on the CP

A three-electrode cell was used to electrochemically deposit a Co hydroxide precursor on a CP in a 0.2 M Co(NO_3_)_2_ aqueous solution. The CP, carbon rod, and Ag/AgCl (saturated KCl) were used as working, counter, and reference electrodes, respectively. The deposition area was controlled to 1 cm^2^ (2 cm × 0.5 cm). The potential of the working electrode was set to −0.7 V *vs.* Ag/AgCl (saturated KCl). After the deposition, the material was sintered under an Ar atmosphere at 300 °C for 3 h to obtain the final product (denoted as Co_3_O_4_/CP).

### Synthesis of CoO_*x*_ on VS_2_/CP

A hydrothermal synthesis was carried out to obtain VS_2_ on the CP (denoted as VS_2_/CP) according to a previous report.^26^ In this synthesis, 0.232 g of ammonium vanadate (NH_4_VO_3_, Nacalai Tesque) and 3.006 g of thioacetamide (CH_3_CSNH_2_, Sigma-Aldrich) were dissolved in 30 mL of deionized water, followed by addition of 2 mL of a 28-mass% ammonium hydroxide (NH_3_·H_2_O, Nacalai Tesque) aqueous solution. The solution was stirred until it turned black, and then transferred to a 50 mL Teflon-lined stainless autoclave container. A CP (5 cm × 0.5 cm) soaked in a 60-mass% nitric acid aqueous solution for more than 24 h was placed in the black solution. The resulting solution was maintained at 160 °C for 20 h. The product was rinsed with water and ethanol several times, followed by drying at 50 °C in air. The Co deposition was carried out according to the procedure used for the synthesis of Co_3_O_4_/CP, except that the CP was replaced with VS_2_/CP. The product is denoted as CoO_*x*_/VS_2_/CP.

### Electrochemical tests

Electrochemical tests were performed using a three-electrode setup. The composite catalyst, Pt wire, and Hg/HgO (0.1 M KOH) were used as the working, counter, and reference electrodes, respectively. Cyclic voltammetry was carried out in an O_2_-saturated 0.1 M KOH aqueous solution to evaluate the OER activity of the composite material. Currents were standardized by the geometric area of the electrode (1 cm^2^). The measured potential, *E* (V *vs.* Hg/HgO), was converted to *E versus* reversible hydrogen electrode (RHE), *E* (V *vs.* RHE), using the equation: *E* (V *vs.* RHE) = *E* (V *vs.* Hg/HgO) + 0.926. Electrochemical impedance spectroscopy (EIS) was carried out to evaluate the impedance of the composite catalysts at 1.6 V *vs.* RHE with an amplitude of 10 mV in the range of 0.1 Hz to 200 kHz.

### Material characterization

The samples were observed by scanning electron microscopy (SEM, JEOL JSM-6010 LA) with energy-dispersive X-ray spectroscopy (EDS). The valence states of the elements on the surfaces of the composite catalysts were measured using X-ray photoelectron spectroscopy (XPS, ULVAC-PHI Quantera). Curve fitting of the XPS spectra was carried out with the Multipak software using a Shirley background function. The structures of the materials were analyzed using a Rigaku UltimalV/285/DX/MO X-ray diffractometer equipped with a Mo K_α_ source (*λ* = 0.7107 Å).

## Conclusions

In this study, we propose a feasible method to obtain cobalt oxide catalysts directly formed through electrochemical deposition and sintering on CP without addition of conductive agents. The cobalt oxides deposited on the CP had a spinel oxide structure, while the cobalt oxides formed on VS_2_ grown on the CP were amorphous. The layered structure of VS_2_ resulted in the amorphization of cobalt oxides. The amorphous cobalt oxides CoO_*x*_/VS_2_/CP exhibited a higher OER activity, smaller diffusion resistance, and higher stability in the OER than those of the crystalized Co_3_O_4_/CP, likely because the amorphous phase did not have grain boundaries that can react or easily dissolve. This study provides a controllable and straightforward method to construct self-supporting cobalt oxide catalysts and elucidates the importance of the crystallinity of cobalt oxides in OER catalysis.

## Conflicts of interest

The authors declare that they have no known competing interests or personal relationships that could have appeared to influence the work reported in this paper.

## Supplementary Material

RA-012-D2RA00492E-s001
