# Supplementary material for: Electrochemical deposition of amorphous cobalt oxides for oxygen evolution catalysis
Source: RSC Adv. 2022 Mar 21;12(14):8731–6. doi: 10.1039/d2ra00492e (PMC8984954; doi:10.1039/d2ra00492e)
Supplement: RA-012-D2RA00492E-s001 [file RA-012-D2RA00492E-s001.pdf]

Electronic Supplementary Material (ESI) for RSC Advances.
This journal is © The Royal Society of Chemistry 2022

Supporting information for

# Electrochemical Deposition of Amorphous Cobalt Oxides for Oxygen Evolution Catalysis

Wei Liu[a], Masao Kamiko[a], Ikuya Yamada[b], Shunsuke Yagi[a,*]

[a]Institute of Industrial Science, The University of Tokyo, 4–6–1 Komaba, Meguro-ku, Tokyo 153–8505, Japan
[b]Department of Materials Science, Graduate School of Engineering, Osaka Prefecture University, 1–2 Gakuen-cho, Naka-ku, Sakai, Osaka 599–8531, Japan

*Corresponding author
Email address: syagi@iis.u-tokyo.ac.jp (S. Yagi)

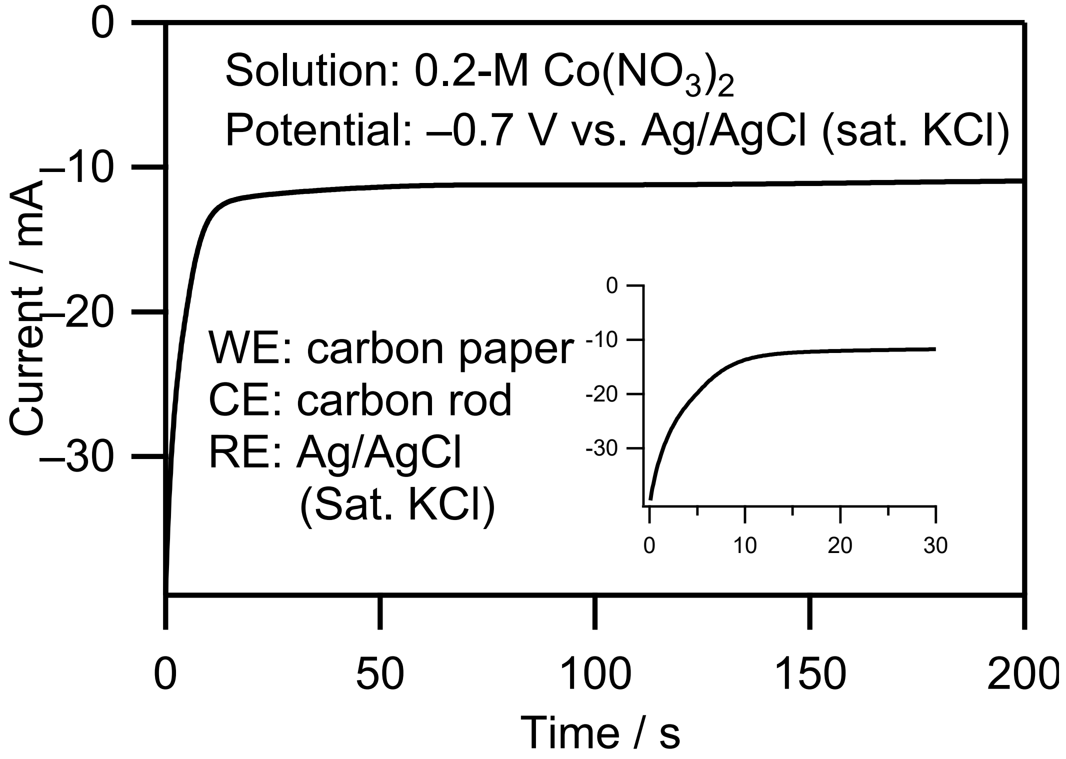


Figure S1 Chronoamperogram during the electrodeposition of $Co_3O_4$ on the CP in a 0.2-M $Co(NO_3)_2$ aqueous solution at −0.7 V vs. Ag/AgCl (saturated KCl).

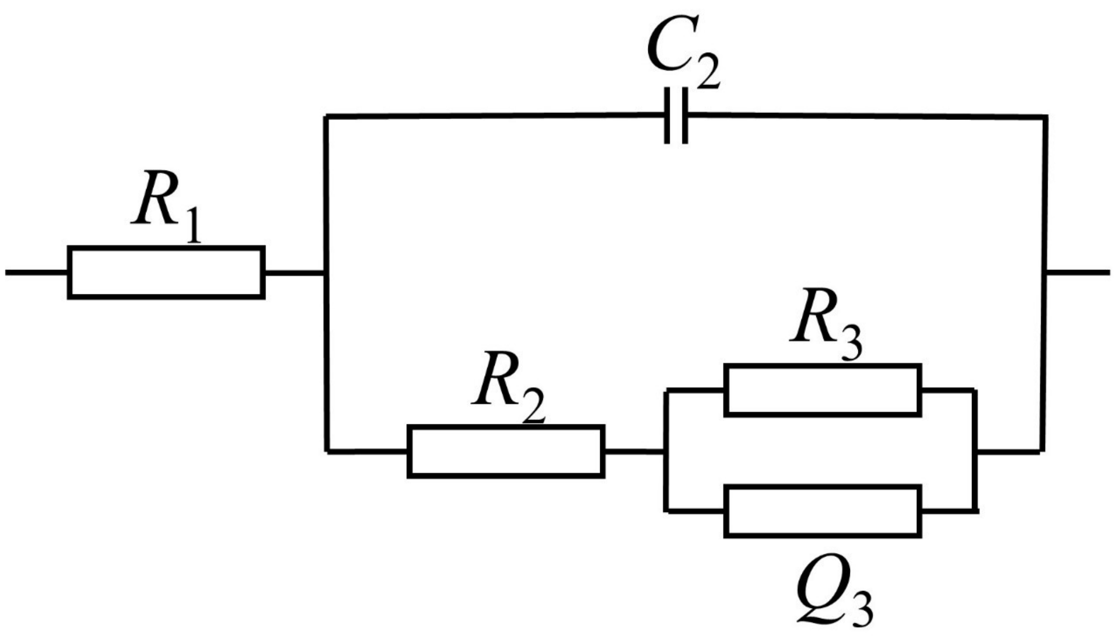


Figure S2 Equivalent circuit used in curve fittings of EIS spectra displayed in Fig. 3b and Fig. 5c.

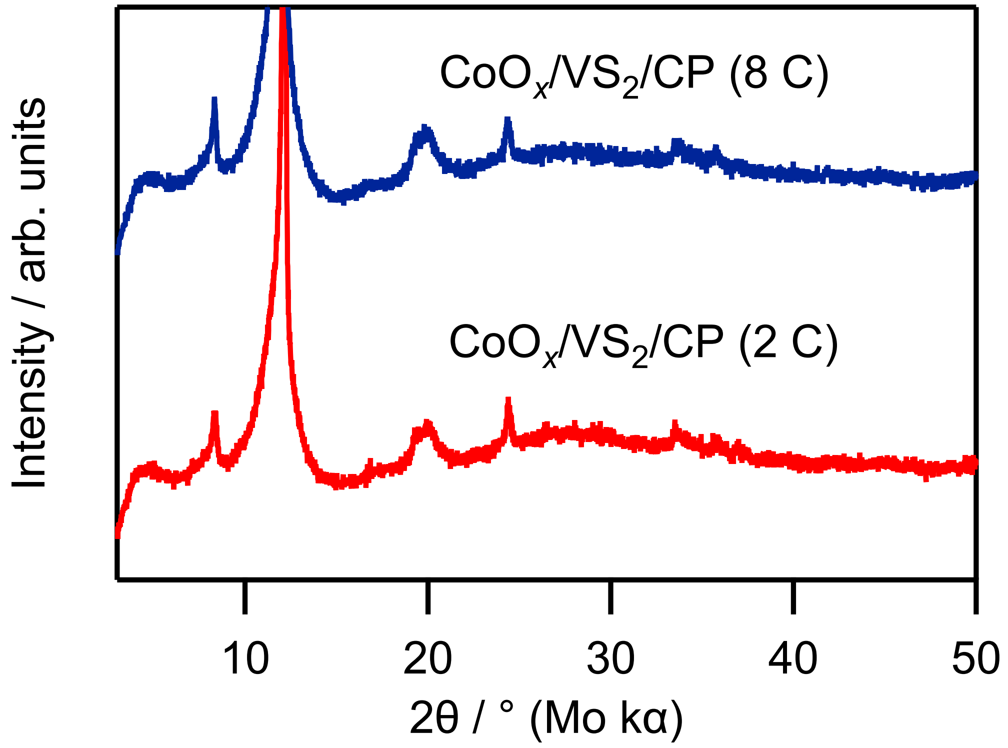


Figure S3 XRD patterns of CoO$_x$/VS$_2$/CP (2 C) and CoO$_x$/VS$_2$/CP (8 C) acquired with a Mo target (0.7107 Å).

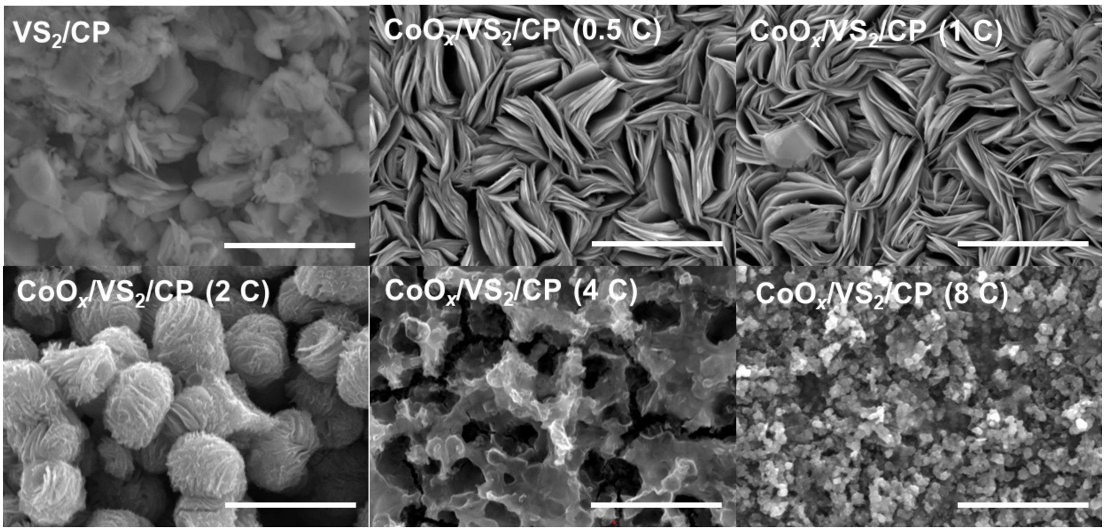


Figure S4 SEM images of $VS_2$/CP and $CoO_x$/$VS_2$/CP with different Co deposition amounts. The scale bar corresponds to 10 µm.

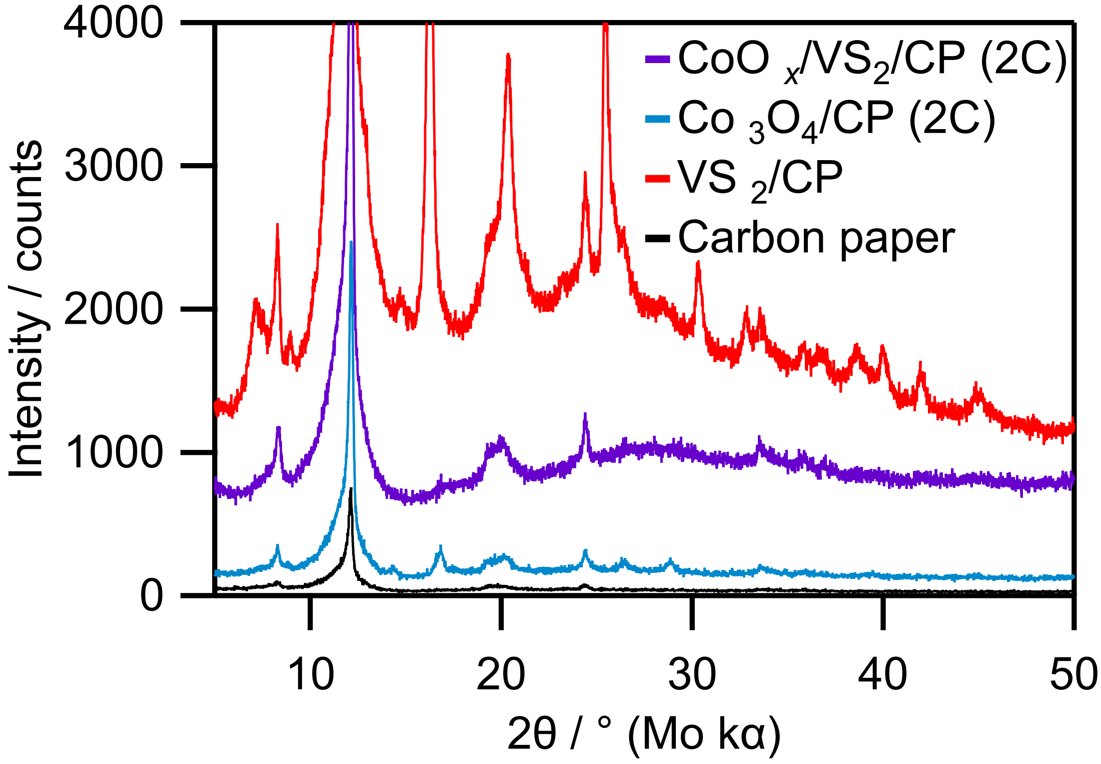


Figure S5 XRD patterns of the synthesized catalysts acquired with the Mo target (0.7107 Å).

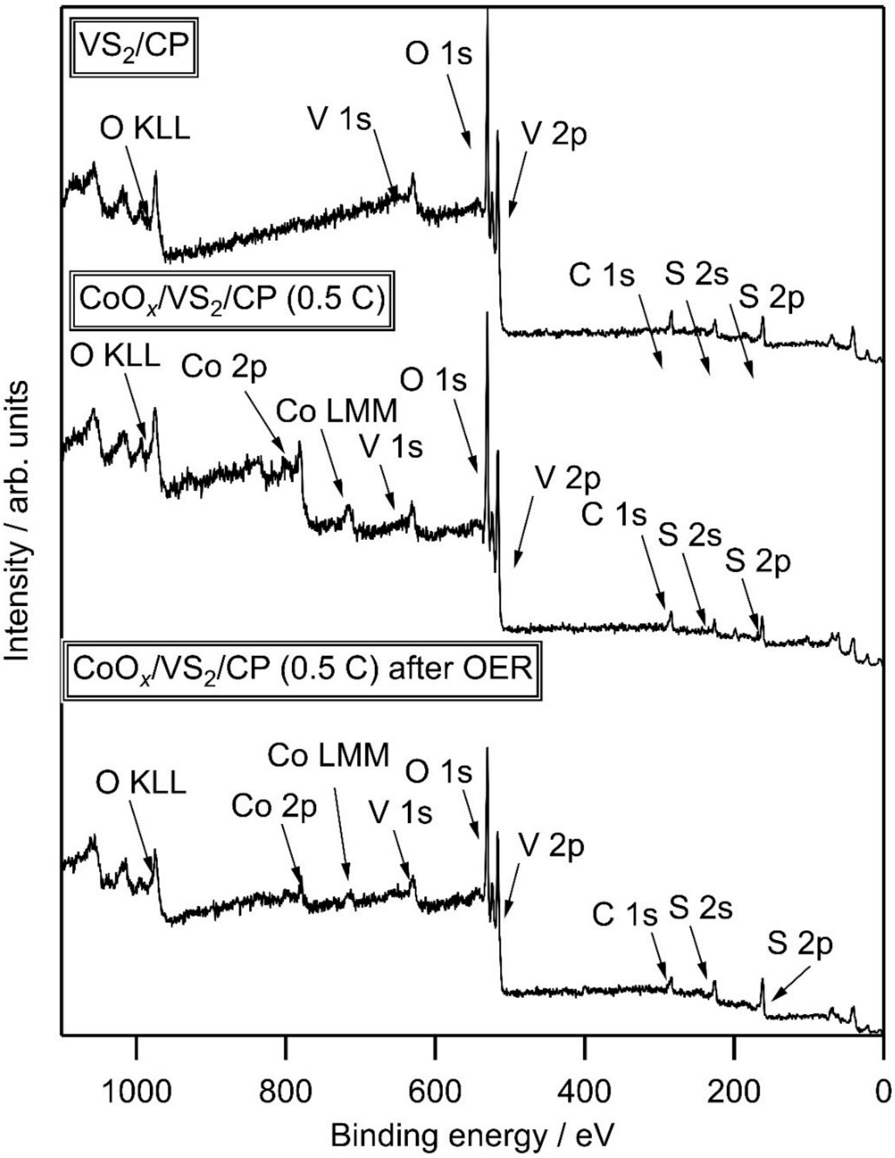


Figure S6 Full XP spectra of $VS_2$/C and $CoO_x$/$VS_2$/CP (0.5 C) before and after the cyclic voltammetry in the range of 1.23 to 1.83 V vs. RHE at a scan rate of 10 mV/s for 100 cycles.

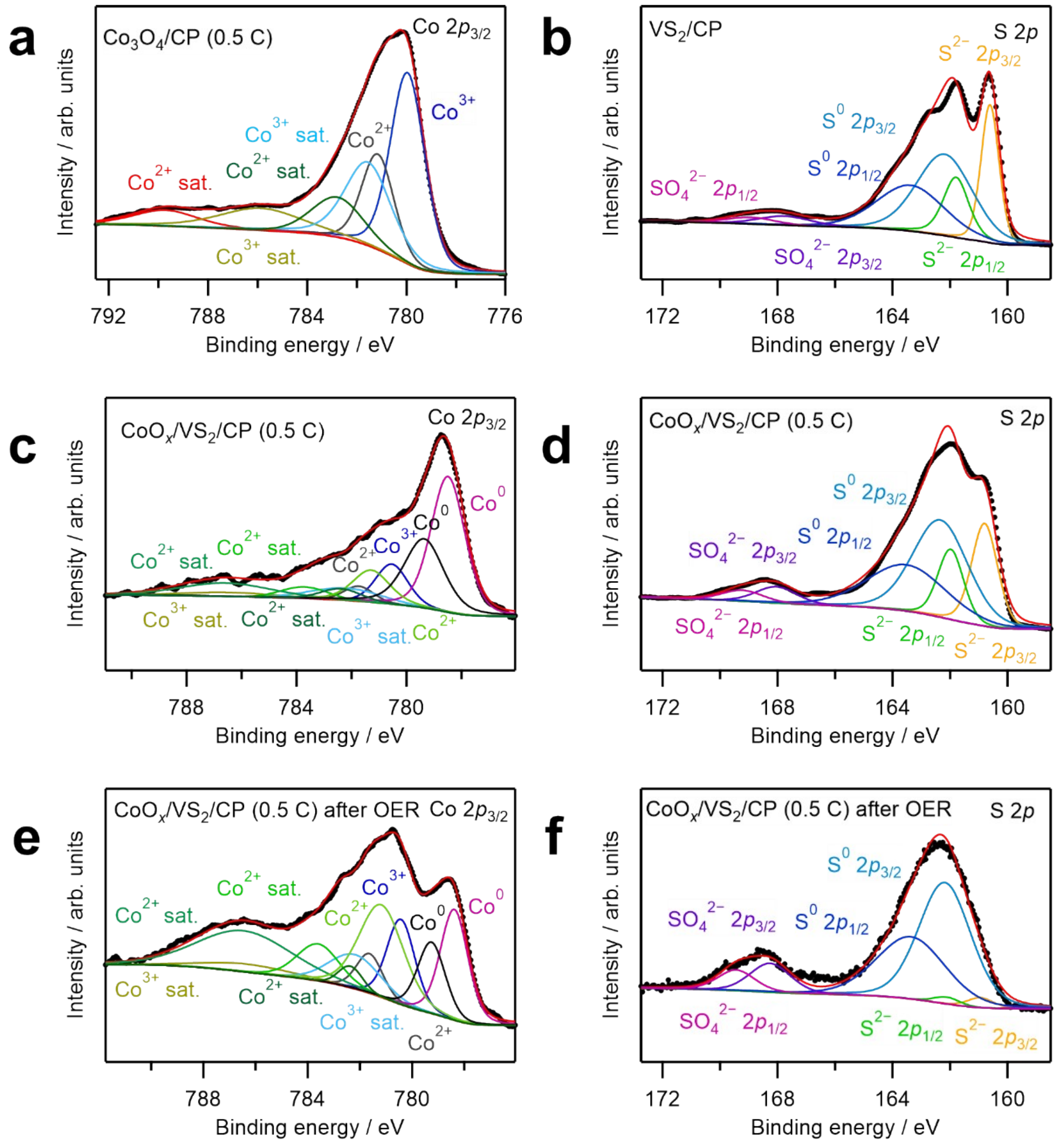


Figure S7 Deconvoluted XP spectra in the Co $2p_{3/2}$ and S $2p$ regions of $Co_3O_4$/CP (0.5 C), $VS_2$/CP, and $CoO_x/VS_2$/CP (0.5 C) before and after the cyclic voltammetry in the range of 1.23 to 1.83 V vs. RHE at a scan rate of 10 mV/s for 100 cycles[1,2].

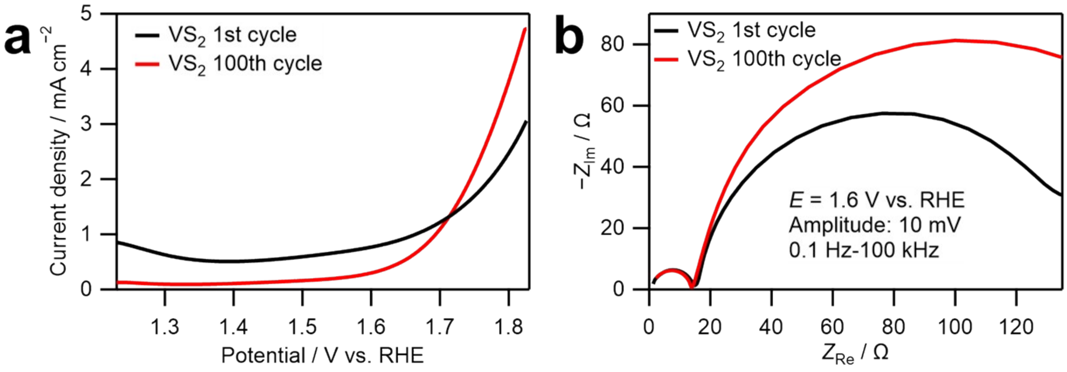


Figure S8 (a) Cyclic voltammograms and (b) Nyquist plots of EI spectra measured for $VS_2$ at the 1st cycle and 100th cycle.

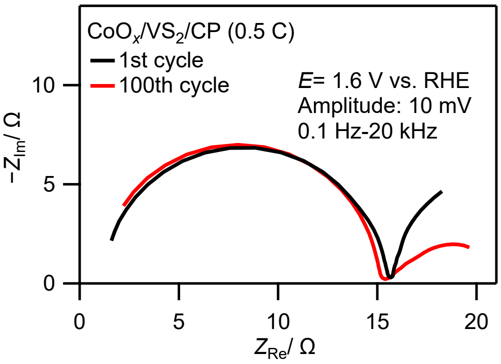


Figure S9 Nyquist plots of EI spectra measured for $CoO_x/VS_2$/CP (0.5 C) at the 1st cycle and 100th cycle.

Table S1 FWHMs of the 311 peaks at 16.8° for the deposited cobalt oxides $Co_3O_4$ on the CP synthesized at different parameters.

| Sample | Temperature/°C | Electric quantity/C | Time/h | 2ϑ/° | FWHM/° |
|---|---|---|---|---|---|
| $Co_3O_4$/CP | 200 | 0.1 | 1 | 16.8 | 0.427 |
| $Co_3O_4$/CP | 200 | 0.1 | 3 | 16.888 | 0.273 |
| $Co_3O_4$/CP | 200 | 0.2 | 1 | 16.849 | 0.402 |
| $Co_3O_4$/CP | 200 | 0.2 | 3 | 16.919 | 0.225 |
| $Co_3O_4$/CP | 250 | 0.2 | 1 | 16.739 | 0.393 |
| $Co_3O_4$/CP | 300 | 0.2 | 1 | 16.830 | 0.385 |

Table S2 Curve fitting results of the Nyquist plots presented in Fig. 3b.

| Sample | $R_1$/Ω | $R_2$/Ω | $C_2$/μF | $R_3$/Ω | $Q_3$/mF | $\alpha_3$ |
|---|---|---|---|---|---|---|
| $Co_3O_4$/CP (0.5 C) | 1.582 | 14.62 | 0.3264 | 44.87 | 4.355 | 0.7651 |
| $Co_3O_4$/CP (1 C) | 1.633 | 15.97 | 0.3002 | 47.39 | 3.892 | 0.8246 |
| $Co_3O_4$/CP (2 C) | 1.333 | 13.33 | 0.3187 | 22 | 14.01 | 0.7018 |
| $Co_3O_4$/CP (4 C) | 1.364 | 14.64 | 0.313 | 34.64 | 9.078 | 0.7119 |
| $Co_3O_4$/CP (8 C) | 1.391 | 14.21 | 0.3029 | 17.35 | 43.05 | 0.5757 |

Table S3 Curve fitting results of the Nyquist plots presented in Fig. 5b.

| Sample | $R_1$/Ω | $R_2$/Ω | $C_2$/μF | $R_3$/Ω | $Q_3$/mF | $\alpha_3$ |
|---|---|---|---|---|---|---|
| CP | 1.059 | 11.93 | 0.4449 | 2114 | 0.1164 | 0.7963 |
| $VS_2$/CP | 1.122 | 13.6 | 0.3893 | 135.1 | 0.8755 | 0.8864 |
| $Co_3O_4$/CP (0.5 C) | 1.582 | 14.62 | 0.3264 | 44.87 | 4.355 | 0.7651 |
| $CoO_x/VS_2$/CP (0.5 C) | 1.296 | 14.16 | 0.3311 | 9.94 | 197.6 | 1 |

**References**


1. M.C. Biesinger, B.P. Payne, A.P. Grosvenor, L.W.M. Lau, A.R. Gerson, R. St.C. Smart, *Appl. Surf. Sci.*, 2011, **257**, 7, 2717–2730.
2. J. Yang, H. Liu, W.N. Martens, and R.L. Frost, *J. Phys. Chem. C*, 2010, **114**, 1, 111–119.
